# Use of airway pressure-based indices to detect high and low inspiratory effort during pressure support ventilation: a diagnostic accuracy study

**DOI:** 10.1186/s13613-023-01209-7

**Published:** 2023-11-13

**Authors:** Yan-Lin Yang, Yang Liu, Ran Gao, De-Jing Song, Yi-Min Zhou, Ming-Yue Miao, Wei Chen, Shu-Peng Wang, Yue-Fu Wang, Linlin Zhang, Jian-Xin Zhou

**Affiliations:** 1 Department of Critical Care Medicine, Beijing Tiantan Hospital, Capital Medical University, Beijing, China; 2 Clinical and Research Center on Acute Lung Injury, Beijing Shijitan Hospital, Capital Medical University, Beijing, 100038 China; 3 Surgical Intensive Care Unit, Beijing Shijitan Hospital, Capital Medical University, Beijing, China; 4 Surgical Intensive Care Unit, China-Japan Friendship Hospital, Beijing, China

**Keywords:** Mechanical ventilation, Inspiratory effort, Airway pressure, Monitoring, Diagnostic test

## Abstract

**Background:**

Assessment of the patient’s respiratory effort is essential during assisted ventilation. We aimed to evaluate the accuracy of airway pressure (*P*_aw_)-based indices to detect potential injurious inspiratory effort during pressure support (PS) ventilation.

**Methods:**

In this prospective diagnostic accuracy study conducted in four ICUs in two academic hospitals, 28 adult acute respiratory failure patients undergoing PS ventilation were enrolled. A downward PS titration was conducted from 20 cmH_2_O to 2 cmH_2_O at a 2 cmH_2_O interval. By performing an end-expiratory airway occlusion maneuver, the negative *P*_aw_ generated during the first 100 ms (*P*_0.1_) and the maximal negative swing of *P*_aw_ (∆*P*_occ_) were measured. After an end-inspiratory airway occlusion, *P*_aw_ reached a plateau, and the magnitude of change in plateau from peak *P*_aw_ was measured as pressure muscle index (PMI). Esophageal pressure was monitored and inspiratory muscle pressure (*P*_mus_) and *P*_mus_–time product per minute (PTP_mus_/min) were used as the reference standard for the patient’s effort. High and low effort was defined as *P*_mus_ > 10 and < 5 cmH_2_O, or PTP_mus_/min > 200 and < 50 cmH_2_O s min^−1^, respectively.

**Results:**

A total of 246 levels of PS were tested. The low inspiratory effort was diagnosed in 145 (59.0%) and 136 (55.3%) PS levels using respective *P*_mus_ and PTP_mus_/min criterion. The receiver operating characteristic area of the three *P*_aw_-based indices by the respective two criteria ranged from 0.87 to 0.95, and balanced sensitivity (0.83–0.96), specificity (0.74–0.88), and positive (0.80–0.91) and negative predictive values (0.78–0.94) were obtained. The high effort was diagnosed in 34 (13.8%) and 17 (6.9%) support levels using *P*_mus_ and PTP_mus_/min criterion, respectively. High receiver operating characteristic areas of the three *P*_aw_-based indices by the two criteria were found (0.93–0.95). A high sensitivity (0.80–1.00) and negative predictive value (0.97–1.00) were found with a low positive predictive value (0.23–0.64).

**Conclusions:**

By performing simple airway occlusion maneuvers, the *P*_aw_-based indices could be reliably used to detect low inspiratory efforts. Non-invasive and easily accessible characteristics support their potential bedside use for avoiding over-assistance. More evaluation of their performance is required in cohorts with high effort.

**Supplementary Information:**

The online version contains supplementary material available at 10.1186/s13613-023-01209-7.

## Background

During assisted ventilation, it is crucial to match the ventilator’s support with the patient’s demand [1]. Both under- and over-assistance may be detrimental [2]. In patients with acute hypoxic respiratory failure, under-assistance may induce vigorous inspiratory effort, leading to increased lung stress and strain, both global and regional, which may potentially result in patient self-inflicted lung injury [3]. On the other hand, over-assistance or deep sedation may result in decreased respiratory drive and low inspiratory effort, thereby prolonging the duration of mechanical ventilation [4]. Extremely high and low effort are also related to diaphragmatic myotrauma, respectively, due to excessive and insufficient muscle loading, which are considered the two hypothetical mechanisms of ventilator-induced diaphragm dysfunction [5, 6]. Therefore, monitoring of inspiratory effort is essential and may facilitate lung and diaphragmatic protection during assisted ventilation.

In daily clinical practice, breathing patterns are usually used to infer increased respiratory workload, such as respiratory distress, the use of accessory inspiratory muscles, and discomfort, but these signs are not quantitative measurements of inspiratory effort [1]. Additionally, these clinical signs usually cannot be used to reliably detect low inspiratory effort induced by over-assistance, because most over-assisted patients appear calm and relaxed. Although variables derived from esophageal pressure (*P*_es_) have been used as a standard reference to evaluate inspiratory effort [7, 8], *P*_es_ monitoring is often employed for research purposes because of invasive procedures with the need for special equipment and complex computations of the effort-associated variables [9, 10]. An easily accessible method is required for the routine effort assessment at the bedside.

Up to now, several indices based on airway pressure (*P*_aw_) analysis have been introduced to assess the inspiratory effort during assisted ventilation [7, 8]. By performing an end-expiratory airway occlusion, the negative *P*_aw_ generated during the first 100 ms (airway occlusion pressure, *P*_0.1_) [11, 12] and the maximal negative swing of *P*_aw_ (∆*P*_occ_) [13, 14] can be easily obtained. In patients undergoing pressure support (PS) ventilation, *P*_aw_ will reach a plateau after end-inspiratory occlusion, and the magnitude of change in plateau from peak *P*_aw_ is defined as pressure muscle index (PMI) [15, 16]. Studies have shown that these *P*_aw_-based indices are associated with *P*_es_-derived effort variables [11–17]. In addition, diagnostic tests have shown that both *P*_0.1_ and ∆*P*_occ_ can accurately identify high [11–14] and low inspiratory effort [12, 17]. Apart from the monitoring of respiratory muscle effort, a recent study has also shown that *P*_0.1_ and ∆*P*_occ_ are accurate in detecting extremes of diaphragmatic effort [17]. However, up to now, studies evaluating the diagnostic performance of PMI for inspiratory effort assessment are limited. Additionally, studies comparing all three *P*_aw_-based indices in the same cohort are also limited.

In the present study, we prospectively enrolled acute respiratory failure patients undergoing PS ventilation. *P*_es_ was monitored and used as the reference for inspiratory effort assessment. The three above-mentioned *P*_aw_-based indices were measured, and the primary objective was to quantify the accuracy of PMI to detect high and low inspiratory efforts.

## Methods

This prospective diagnostic accuracy study was conducted in four ICUs of two academic hospitals (Beijing Tiantan Hospital and Beijing Shijitan Hospital, Capital Medical University, Beijing, China). The protocol was approved by the respective Institutional Review Board of the two hospitals (No. KY2021-012-01 and No. SJTKY-ER-2023-38). Written informed consent was obtained from the patient or their legal representative. The study design, performance, and report were compliant with the Standards for Reporting of Diagnostic Accuracy (STARD) guidelines [18].

### Patients

Adult acute hypoxic respiratory failure patients undergoing mechanical ventilation were screened daily and enrolled within 24 h after the transition from controlled ventilation to PS mode. During the study, the patients were consecutively recruited. In the study units, acute hypoxic respiratory failure was diagnosed as acute shortness of breath and hypoxemia which was defined as the partial pressure of oxygen in arterial blood (PaO_2_) lower than 60 mmHg at room air or PaO_2_/inspired oxygen fraction (FiO_2_) lower than 300 mmHg. There was no formal protocol for mechanical ventilation to guide the transit from the controlled mode to an assisted mode in the participating units so the transition was at the discretion of the ICU physician team. Analgesia was routinely used in mechanically ventilated patients with fentanyl or remifentanil. Sedation with propofol or dexmedetomidine was used when the patient exhibited agitation and a light sedation level was maintained (Richmond Agitation Sedation Scale of − 1 to + 1).

Exclusion criteria included: (1) age younger than 18 years old; (2) history of neuromuscular diseases; (3) history of diaphragm dysfunction and surgery; (4) history of esophageal, gastric, or lung surgery; (5) history of chronic obstructive pulmonary diseases; (6) decreased level of consciousness (defined by the motor response of Glasgow Coma Scale ≤ 4); (7) central respiratory drive dysfunction (defined by irregular breathing patterns due to brain stem lesions including tumor, trauma, and stroke); and (8) considered withholding of life support. The patients with COVID-19 were also excluded.

### Protocol and data collection

After enrollment, an esophageal balloon catheter (Cooper catheter: LOT 177405, Cooper Surgical, USA) was placed as the method described previously, and an occlusion test was performed to confirm the proper balloon position [9, 10]. The ventilators used in the present study included Dräger V500 (Dräger, Lubeck, Germany), Maquet Servo-i (Maquet Critical Care, Solna, Sweden), and Prunus Padus 8 (Prunus Medical, Shenzhen, China).

During the procedure, patients remained in a supine position with the head of bed elevated to 30°. Baseline data were collected at clinical ventilator settings adjusted by the responsible ICU physicians. Thereafter, with the FiO_2_, trigger sensitivity, positive end-expiratory pressure (PEEP), and cycle-off criteria remaining unchanged, a downward PS titration was performed from 20 cmH_2_O to 2 cmH_2_O at a 2 cmH_2_O interval. Each PS level was maintained for 20 min, and then the airway was occluded by using the ventilator hold function. First, three nonconsecutive short end-expiratory occlusions were performed. Only one inspiratory effort was induced by each occlusion. Thereafter, three nonconsecutive end-inspiratory occlusions were performed with each longer than 2 s. Either end-expiratory or end-inspiratory occlusions were performed with 60-s intervals between them. The three measurements of either end-expiratory occlusion or end-inspiratory occlusion at each PS level were averaged to one value, which was then used for the analysis.

The experimental procedures were performed by one investigator in each participating ICU who was trained before the start of the study. The following key points were emphasized to guarantee the quality of collected data [19]: (1) check the air leak before the initiation of each tested PS level, including cuff check and observation of inspiratory and expiratory tidal volume difference; (2) observe the flow-time waveform during occlusion (maintaining zero flow); (3) induce only one inspiratory effort by each end-expiratory occlusion; (4) a longer than a 2-s duration of end-inspiratory occlusion; and (5) observe the plateau *P*_aw_ during the end-inspiratory occlusion (flat shape).

During downward PS titration, the test was stopped if the patient showed signs of respiratory distress, which were defined as (1) heart rate of more than 140 beats/min; (2) increase in respiratory rate by 50% or more; (3) hypotension (systolic blood pressure lower than 90 mm Hg) or hypertension (systolic blood pressure higher than 160 mm Hg); (4) cardiac arrhythmia; (5) peripheral arterial oxygen saturation lower than 90%; (6) use of accessory respiratory muscles, diaphoresis, agitation, and the appearance of abdominal or thoracic paradoxical movements [20]. If apnea appeared at high PS levels which was indicated by the initiation of backup ventilation, the PS was decreased to the next lower level.

Flow, *P*_aw_, and *P*_es_ data were collected using a heated Fleisch pneumotachograph (Vitalograph Inc, Lenexa, KS, USA) and two pressure transducers (KleisTEK Engineering, Bari, Italy). Signals were displayed continuously and saved on a laptop for offline analysis, at a sample rate of 200 Hz (ICU-Lab 2.5 Software Package, KleisTEK Engineering, Bari, Italy).

### Inspiratory effort measurements

**Fig. 1:**
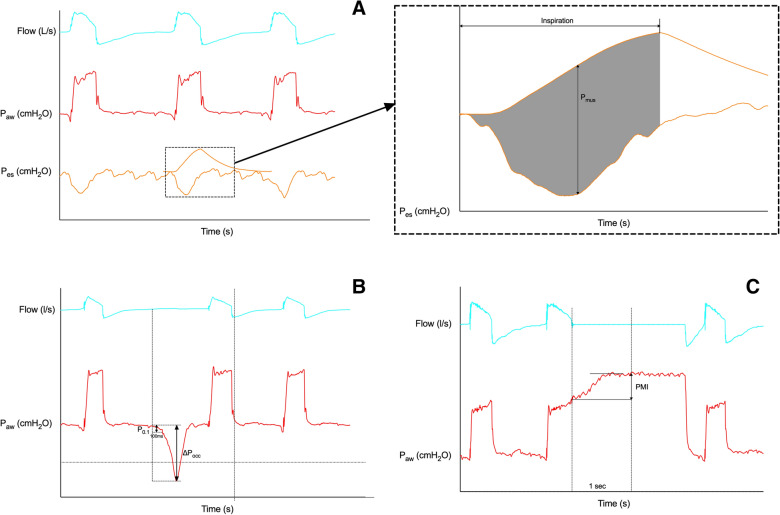
Offline analyses of inspiratory effort using the total respiratory muscle pressure–time product (**A**), the negative airway pressure generated during the first 100 ms (*P*_0.1_) and the maximal negative swing of airway pressure (∆*P*_occ_) against an end-expiratory airway occlusion (**B**), and pressure muscle index (PMI) induced by an end-inspiratory airway occlusion (**C**). *P*_aw_ airway pressure, *P*_es_ esophageal pressure, *P*_mus_ pressure generated by respiratory muscle during inspiration

Offline analyses of flow-, *P*_aw_-, and *P*_es_-time waveforms, which are schematically shown in Fig. 1, were performed independently by two investigators (YL and RG). When the two measurements were discrepant, a group discussion was held with two other senior investigators (YLY and YMZ) to arrive at a consensus.

#### *P*_es_-derived inspiratory effort measurements

For measurements of *P*_es_-derived inspiratory effort variables, breathings without *P*_es_ artifacts and patient–ventilator asynchrony were identified within 1 min prior to the performance of airway occlusions [21]. Then the measured values were averaged.

The onset of inspiratory effort was defined as the point of negative deflection of *P*_es_ with a rapid change in slope [15]. We defined the start and the end of ventilator insufflation as the respective point of flow zero-crossing. Intrinsic PEEP was measured as the absolute change in *P*_es_ from the onset of inspiratory effort to the start of ventilator insufflation [12, 22]. The pressure generated by respiratory muscle during inspiration (*P*_mus_) was calculated as the maximal difference between the *P*_es_ and quasi-static recoil pressure of the chest wall [9, 10], which was constructed by the measured chest wall elastance using the difference between plateau *P*_es_ induced by end-inspiratory airway occlusion and end-expiratory *P*_es_ [15, 16]. Data at pressure support of 20 cmH_2_O (or the highest support level during titration) were used to calculate chest wall elastance because the patient’s inspiratory effort was minimal at high support levels. *P*_mus_–time product (PTP_mus_) per breath was calculated as the time-integral of the *P*_mus_, from the onset of inspiratory effort to the end of ventilator insufflation (Fig. 1A). PTP_mus_ per minute was calculated as the product of the averaged PTP_mus_ per breath times respiratory rate.

#### *P*_aw_-based inspiratory effort indices

*P*_aw_-based inspiratory effort indices were measured and averaged from the three end-inspiratory and end-expiratory airway occlusion maneuvers.

Against an end-expiratory airway occlusion, *P*_0.1_ and ∆*P*_occ_ were measured as the drop of *P*_aw_ from the onset of inspiratory effort until 100 ms [22, 23] and the maximal decline in *P*_aw_ from PEEP [13, 14], respectively (Fig. 1B).

After the onset of end-inspiratory airway occlusion, the *P*_aw_ reached a plateau, and PMI was measured as the difference between the peak *P*_aw_ (*P*_aw_ just before the end-inspiratory occlusion indicated by the onset of zero-flow) and plateau *P*_aw_ (1 s after the occlusion) (plateau–peak) (Fig. 1C) [15, 16].

**Fig. 2:**
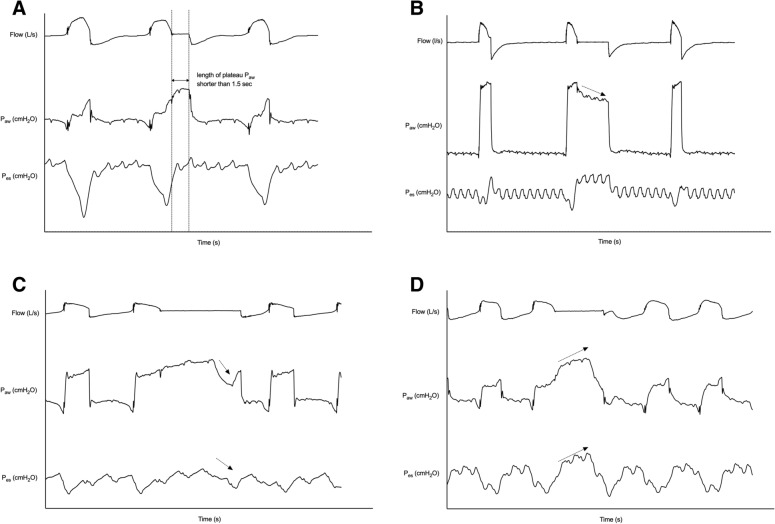
Example of discarded measurement of pressure muscle index during offline analysis. **A** Too short plateau airway pressure (*P*_aw_) during end-inspiratory occlusion (set a priori as shorter than 1.5 s); **B** presence of air leak, which is indicated by a continued decrease in *P*_aw_ without reaching a plateau simultaneous with a relatively stable plateau esophageal pressure (*P*_es_) during end-inspiratory occlusion; **C** suspicion of additional inspiratory effort. During the end-inspiratory occlusion, additional inspiratory effort is suspected by the simultaneous decrease of *P*_aw_ and *P*_es_ (arrows); **D** suspicion of additional expiratory effort. During the end-inspiratory occlusion, additional expiratory effort was suspected by the increase in *P*_aw_ and *P*_es_ (arrows)

Because end-inspiratory occlusion during PS ventilation may result in an unstable plateau *P*_aw_ [16, 24], we paid special attention to the measurement of PMI. We set a priori to discard measurements, including a length of plateau shorter than 1.5 s, the presence of air leak, and suspicion of additional effort during the occlusion (Fig. 2) [16]. The reasons for excluded measurements were reported.

### Definition of high and low inspiratory effort

PTP_mus_ per minute and *P*_mus_ was used as the reference for inspiratory effort measurement. For PTP_mus_ per minute, low, intermediate, and high effort was pre-defined as < 50, 50–200, and > 200 cmH_2_O s min^−1^, respectively [7, 8, 12]. For *P*_mus_, low, intermediate, and high effort was pre-defined as < 5, 5–10, and > 10 cmH_2_O, respectively [13, 17, 25].

### Statistical analysis

Continuous variables are presented as the median and interquartile range (IQR) and categorical variables as numbers and percentages. The comparison of variables among different PS levels, as well as among different classifications of inspiratory effort, was performed using the Kruskal–Wallis test, followed by pairwise post hoc analysis with Dunn’s correction.

With patients as a random effect and PS levels as repeated measures, the association of each of the three *P*_aw_-based indices (*P*_0.1_, ∆*P*_occ_, and PMI) with respective PTP_mus_ per minute and *P*_mus_ was analyzed using linear mixed-effects regression models.

The diagnostic accuracy of tested *P*_aw_-based indices to detect low and high effort was analyzed using the receiver operating characteristics (ROC) curve. The area under the curve (AUC) and 95% confidence interval (CI) were calculated. A comparison of AUC was performed using the DeLong test. The best cutoff values were identified by the Youden index. Sensitivity, specificity, and positive and negative predictive values (PPV and NPV) were calculated using the standard formula. Tenfold cross-validation was used to observe the stability of the diagnostic performance.

In order to test the clinical impact of the three *P*_aw_-based indices, we, respectively, calculated upper (90%) and lower (10%) limits of reference intervals, according to the guidance published by the Clinical and Laboratory Standards Institute [26]. The median and 95% CI of upper/lower limits of reference intervals were estimated. The percentages of conditions lying in the "grey zone", which was defined as the condition outside of the lower to upper limit range of reference intervals, were also counted.

PMI was our primary variable of interest. For the sample size calculation, we estimated that PMI would have at least acceptable diagnostic accuracy to detect high inspiratory effort with an AUC of 0.80. A previous study reported that the occurrence of high effort was approximately 10% during PS titration [25]. Therefore, 150 levels of PS were required to obtain a Type I error of 0.05 and a Type II error of 0.80 to construct an ROC analysis. Considering that 10% of pressure support levels could not be tolerated during the titration [20], as well as nearly 40% of offline PMI measurements could not be performed due to inappropriate plateau *P*_aw_ waveform [16], 280 PS levels, namely 28 patients, would be needed.

Statistical analyses were performed using the MedCalc (2022 MedCalc Software Ltd, Belgium) and R4.1.2 (www.R-project.org). A *P*-value lower than 0.05 was regarded as statistically significant.

## Results

Clinical characteristics of enrolled patients (*n* = 28) are shown in Table 1. During the study, 22 patients fulfilled all tested PS levels without interruption. Titration was stopped at PS of 4 cmH_2_O in three patients due to respiratory distress. Because of the occurrence of apnea at high support levels, tests were started at 18 and 16 cmH_2_O in one and two patients, respectively. Therefore, 269 PS levels were used for offline analysis.

**Table 1:** Patients’ characteristics

Variables	*n* = 28
Male, *n* (%)	16 (57%)
Age (years)	59 (48, 67)
Body mass index (kg/m^2^)	24.7 (22.6, 26.9)
Diagnosis	
Pneumonia	9 (32%)
Sepsis	7 (25%)
Trauma	7 (25%)
Postoperative	5 (18%)
APACHE II	20 (16, 22)
Mechanical ventilation days before inclusion (days)	7 (4, 10)
Mechanical ventilation settings at enrollment	
PS (cmH_2_O)	8 (8, 10)
PEEP (cmH_2_O)	5 (5, 5)
FiO_2_	0.40 (0.35, 0.40)
Ventilation parameters at enrollment	
Tidal volume (ml)	538 (456, 618)
Respiratory rate (breaths/min)	17 (14, 19)
Minute ventilation (L/min)	8.5 (7.0, 11.2)
Blood gas at enrollment	
PaO_2_ (mmHg)	121 (85, 135)
PaO_2_/FiO_2_	313 (213, 381)
PaCO_2_ (mmHg)	38.4 (34.3, 41.7)
Respiratory mechanics	
Pmus (cmH2O)	5.0 (2.8, 6.7)
PTPmus (cmH2O s min^−1^)	55.9 (31.9, 79.9)
Ers	16.1 (13.7, 18.7)
Elung	3.8 (2.9, 5.4)
Ecw	11.2 (9.3, 14.5)
PEEPi	1 (0, 1)
*P*_0.1_ (cm H_2_O)	1.6 (0.4, 2.1)
∆*P*_occ_ (cm H_2_O)	6.8 (3.1, 9.9)
PMI (cm H_2_O)	0.4 (− 0.8, 1.5)
Sedation	10 (38%)
RASS	0 (− 1, 0)
Ventilator-free days	4 (2, 7)
Length of stay in ICU (days)	19 (12, 33)
Length of stay in hospital (days)	29 (16, 41)
Hospital mortality	3 (10.7%)

Data are shown as median (interquartile range) or *n* (%)

FiO_2_: inspired oxygen fraction; PaCO_2_: partial pressure of carbon dioxide in arterial blood; PaO_2_: partial pressure of oxygen in arterial blood; PEEP: positive end-expiratory pressure; PS: pressure support; APACHE II: Acute Physiology and Chronic Health Evaluation II; *P*_0.1_: negative swing of airway pressure against end-expiratory airway occlusion at first 100 ms; ∆*P*_occ_ the maximal negative swing of airway pressure against end-expiratory airway occlusion; PMI: pressure muscle index; RASS: Richmond Agitation Sedation Scale

### PMI measurement

After inspecting *P*_aw_ waveforms during end-inspiratory occlusion, measurements of PMI at 23 (8.6%) PS levels were excluded due to suspicion of air leaks (3 levels, 13.0%) at high PS levels, the length of plateau shorter than 1.5 s (2 levels, 8.7%), and inspiratory (12 levels, 52.2%) and expiratory (6 levels, 26.1%) effort during occlusion at low PS levels (Fig. 2). Finally, data at 246 PS levels were measured and used for analysis.

### Correlation between *P*_aw_-based indices and *P*_es_-derived variables

**Fig. 3:**
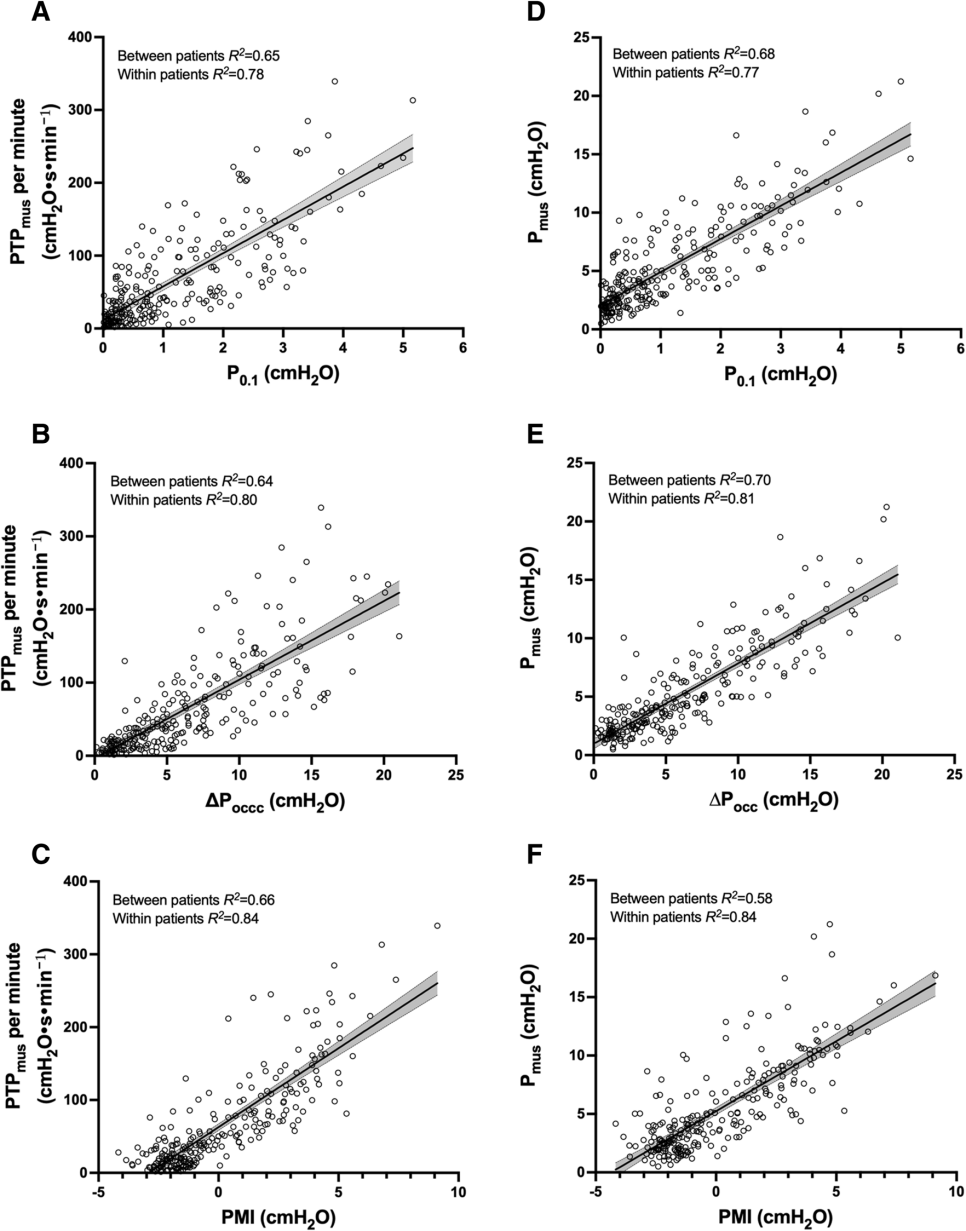
The negative airway pressure generated during the first 100 ms (*P*_0.1_), the maximal negative swing of airway pressure (∆*P*_occ_) against an end-expiratory airway occlusion, and pressure muscle index (PMI) significantly correlated with the inspiratory muscle pressure–time product (PTP_mus_) per minute (**A**–**C**) and the inspiratory muscle pressure (*P*_mus_) (**D**–**F**). The results of linear mixed-effects regression, regression line, and corresponding 95% confidence interval (shaded grey area) are shown

Downward PS adjustment resulted in a significant change in PTP_mus_ per minute and *P*_mus_, as well as *P*_aw_-based indices (*P* < 0.001) (Additional file 1**:** Fig. S1). A significant correlation was found between the *P*_aw_-based indices with PTP_mus_ per minute (between-patients *R*^*2*^ = 0.64–0.65; within-patients *R*^*2*^ = 0.78–0.84) and *P*_mus_ (between-patients *R*^*2*^ = 0.58–0.70; within-patients *R*^*2*^ = 0.77–0.84) (Fig. 3).

### Classification of inspiratory effort according to reference standards

**Fig. 4:**
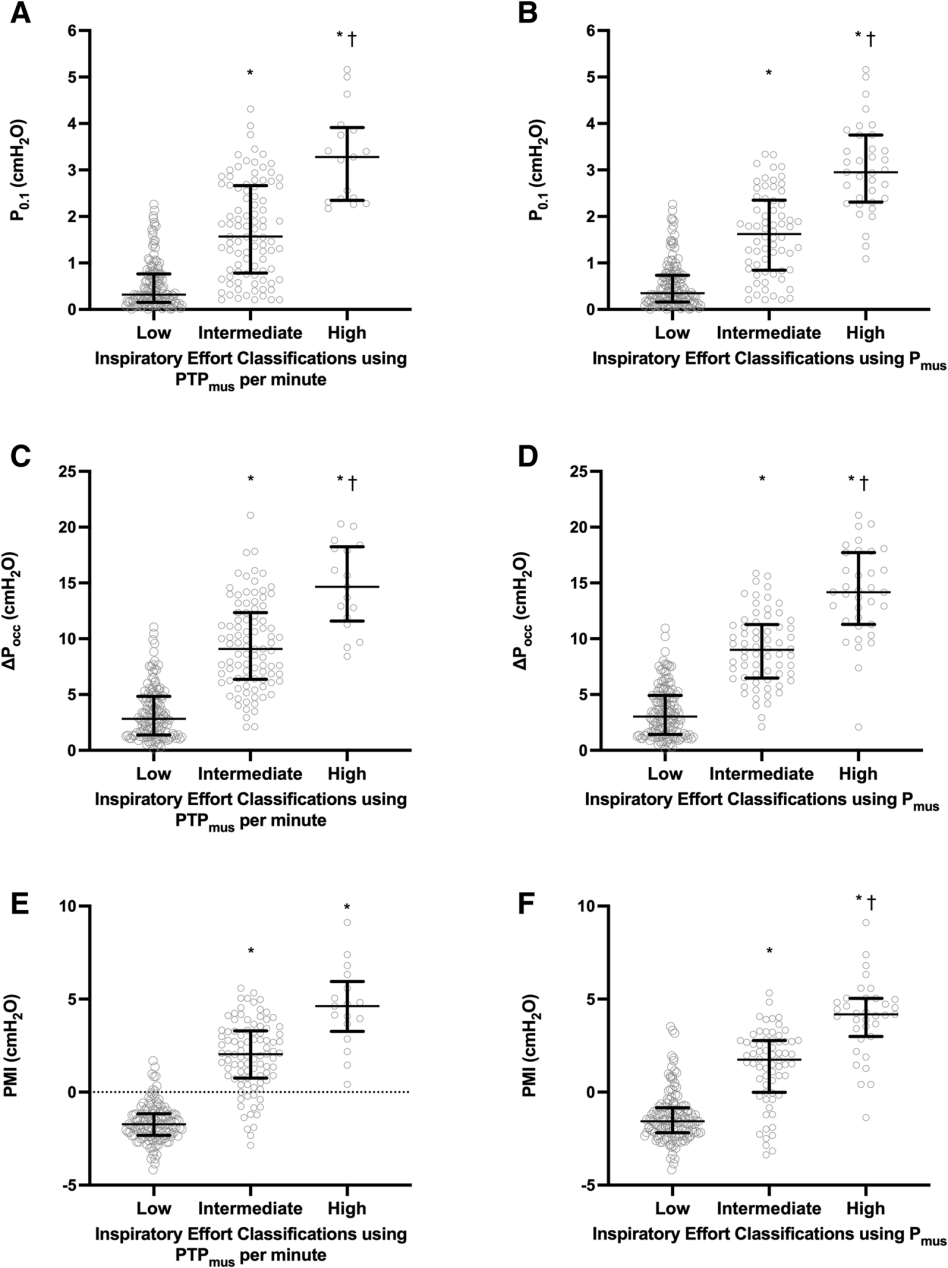
Among low, intermediate, and high inspiratory effort classification groups according to the criteria of inspiratory muscle pressure (*P*_mus_) and *P*_mus_–time product (PTP_mus_) per minute, a significant difference was found in the negative airway pressure generated during the first 100 ms (P_0.1_) against an end-expiratory airway occlusion (**A** and **B**), the maximal negative swing of airway pressure (∆*P*_occ_) against an end-expiratory airway occlusion (**C** and **D**), and pressure muscle index (PMI) (**E** and **F**). Data are shown as median and interquartile range. ^*^Significantly higher than the low effort group, ^†^significantly higher than the intermediate effort group

Using PTP_mus_ per minute as the reference, the low, intermediate, and high inspiratory effort was diagnosed at 136 (55.3%), 93 (37.8%), and 17 (6.9%) PS levels, with respective median (IQR) PS of 14 (12–18), 6 (4–8), and 4 (2–7) cmH_2_O. When *P*_mus_ was used as the reference, 146 (59.3%), 65 (26.5%), and 35 (14.2%) PS levels (median [IQR]: 14 [10–18] vs 8 [4–10] vs 4 [2–6] cmH_2_O) were diagnosed as low, intermediate, and high effort, respectively. Respiratory mechanics and gas exchange in the three groups are shown in Additional file 1: Tables S1 and S2. Among the three effort groups by either PTP_mus_ per minute or *P*_mus_ criterion, significant differences were found in the three *P*_aw_-based indices (Fig. 4) (*P* < 0.001).

### Diagnostic performance for high inspiratory effort

For detecting high inspiratory effort by either PTP_mus_ per minute (> 200 cmH_2_O·s·min^−1^) or *P*_mus_ (> 10 cmH_2_O), *P*_0.1_, ∆*P*_occ_, and PMI showed excellent discriminative accuracy with AUC ranging from 0.93 to 0.95, and no significant difference was found between each paired comparison (Table 2). According to the best cutoff value identified by the Youden index, the three *P*_aw_-based indices showed high sensitivity (0.80 to 1.00) and high NPV (0.97 to 1.00), with a low PPV (0.23 to 0.64).

**Table 2:** Diagnostic performance for detecting high inspiratory effort

Indices	AUC	Cutoff	Sensitivity	Specificity	PPV	NPV
Classified by PTP_mus_ per minute (> 200 cmH_2_O s min^−1^)						
*P*_0.1_ (cmH_2_O)	0.94 (0.91–0.98)	2.2	1.00 (0.80–1.00)	0.86 (0.81–0.90)	0.35 (0.28–0.42)	1.00 (0.98–1.00)
∆*P*_occ_ (cmH_2_O)	0.93 (0.88–0.97)	8.4	1.00 (0.80–1.00)	0.75 (0.69–0.81)	0.23 (0.19–0.27)	1.00 (0.98–1.00)
PMI (cmH_2_O)	0.93 (0.88–0.98)	2.1	0.88 (0.64–0.99)	0.81 (0.76–0.86)	0.26 (0.20–0.32)	0.99 (0.96–1.00)
Classified by *P*_mus_ (> 10 cmH_2_O)						
*P*_0.1_ (cmH_2_O)	0.95 (0.92–0.98)	2.0	0.91 (0.77–0.98)	0.89 (0.84–0.93)	0.57 (0.47–0.66)	0.98 (0.95–0.99)
∆*P*_occ_ (cmH_2_O)	0.93 (0.89–0.98)	9.2	0.94 (0.81–0.99)	0.84 (0.79–0.89)	0.50 (0.42–0.58)	0.99 (0.96–1.00)
PMI (cmH_2_O)	0.93 (0.89–0.98)	2.8	0.80 (0.63–0.92)	0.92 (0.88–0.96)	0.64 (0.52–0.74)	0.97 (0.93–0.98)

95% confidence intervals are shown in parentheses for diagnostic performance measures

PTP_mus_: inspiratory muscle pressure–time product; *P*_0.1_: negative swing of airway pressure against end-expiratory airway occlusion at first 100 ms; *∆P*_occ_: the maximal negative swing of airway pressure against end-expiratory airway occlusion; PMI: pressure muscle index; *P*_mu*s*_: inspiratory muscle pressure; AUC: area under the receiver-operating-characteristics curve; PPV: positive predictive value; NPV: negative predictive value

Tenfold cross-validation yielded the same cutoff values and diagnostic performance parameters for the two references (Additional file 1: Table S3).

### Diagnostic performance for low inspiratory effort

Results of diagnostic performance for detecting low inspiratory effort are shown in Table 3. *P*_0.1_, ∆*P*_occ,_ and PMI showed excellent discriminative accuracy (AUC: 0.87 to 0.95) for low effort by using either of the two criteria (PTP_mus_ per minute < 50 cmH_2_O s min^−1^ and *P*_mus_ < 5 cmH_2_O). By using PTP_mus_ per minute as the criterion, the best cutoff values of *P*_0.1_, ∆*P*_occ,_ and PMI were 1.1, 5.7, and 0 cmH_2_O, respectively. The respective best cutoff value determined by *P*_mus_ criterion was 1.2 cmH_2_O for *P*_0.1_, 6.2 cmH_2_O for ∆*P*_occ_, and 0 cmH_2_O for PMI. The sensitivity and specificity of the three *P*_aw_-base indices ranged from 0.83 to 0.96 and 0.74 to 0.88, with PPV and NPV of 0.80 to 0.91 and 0.78 to 0.94, respectively.

**Table 3:** Diagnostic performance for detecting low inspiratory effort

Indices	AUC	Cutoff	Sensitivity	Specificity	PPV	NPV
Classified by PTP_mus_ per minute (< 200 cmH_2_O s min^−1^)						
*P*_0.1_ (cmH_2_O)	0.87 (0.83–0.92)^a^	1.1	0.88 (0.81–0.93)	0.74 (0.64–0.82)	0.80 (0.75–0.85)	0.83 (0.75–0.88)
∆*P*_occ_ (cmH_2_O)	0.93 (0.90–0.96)	5.7	0.87 (0.80–0.92)	0.85 (0.77–0.91)	0.88 (0.82–0.92)	0.84 (0.77–0.89)
PMI (cmH_2_O)	0.95 (0.92–0.98)	0.0	0.96 (0.91–0.98)	0.86 (0.79–0.92)	0.90 (0.84–0.93)	0.94 (0.88–0.97)
Classified by *P*_mus_ (< 10 cmH_2_O)						
*P*_0.1_ (cmH_2_O)	0.90 (0.87–0.94)	1.2	0.90 (0.84–0.94)	0.78 (0.69–0.86)	0.86 (0.80–0.90)	0.84 (0.76–0.89)
∆*P*_occ_ (cmH_2_O)	0.94(0.91–0.97)^a^	6.2	0.88 (0.81–0.93)	0.86 (0.78–0.92)	0.90 (0.85–0.94)	0.83 (0.75–0.88)
PMI (cmH_2_O)	0.89 (0.85–0.94)	0.0	0.83 (0.76–0.89)	0.88 (0.80–0.94)	0.91 (0.86–0.95)	0.78 (0.71–0.84)

95% confidence intervals are shown in parentheses for diagnostic performance measures

PTP_mus_: inspiratory muscle pressure–time product; *P*_0.1_: negative swing of airway pressure against end-expiratory airway occlusion at first 100 ms; *∆P*_occ_: the maximal negative swing of airway pressure against end-expiratory airway occlusion; PMI: pressure muscle index; *P*_mus_: inspiratory muscle pressure; AUC: area under the receiver-operating-characteristics curve; PPV: positive predictive value; NPV: negative predictive value

^a^Significant difference was found compared to other indices

Tenfold cross-validation revealed the same cutoff values, without significant changes in diagnostic accuracy parameters for the two references (Additional file 1: Table S4).

### Analysis of reference intervals

Results of the median and upper/lower limits of reference intervals with *P*_0.1_, ∆*P*_occ_, and PMI are shown in Additional file 1: Table S5. The percentages of conditions lying in the "grey zone" ranged from 4.3% to 9.7% by PTP_mus_ per minute criterion, and from 3.1% to 12.3% by *P*_mus_ criterion.

## Discussion

In the present study, we comprehensively investigated the diagnostic performance of three *P*_aw_-based indices for inspiratory effort assessment, *P*_0.1_, ∆*P*_occ_, and PMI, which have been introduced in previous clinical investigations. In accordance with previous results of the association of *P*_0.1_ and ∆*P*_occ_ with *P*_es_-derived effort variables [12, 13], we also found a strong correlation between each of the three *P*_aw_-based indices and PTP_mus_ per minute as well as *P*_mus_. For detecting low inspiratory effort, excellent discrimination and balanced diagnostic performance indicated that all these three indices could be reliably used to rule in and rule out the condition. For the detection of high effort by the *P*_aw_-based indices, although high sensitivities and high NPVs were obtained, these results should be interpreted with caution because a very low proportion of high effort was induced in our cohort.

### References for diagnosis of high and low inspiratory effort

In general, three *P*_es_-derived variables provide the criteria for low or high inspiratory effort, including *P*_mus_, PTP_mus_ per minute, and work of breathing [9, 10]. In the present study, we selected PTP_mus_ per minute and *P*_mus_ as the references, which were mostly used in previous studies [12, 13, 17, 25]. *P*_0.1_ is related to respiratory drive and work of breathing [12, 17], thus PTP_mus_ per minute would be a good reference to define the extremes of effort. However, because ∆*P*_occ_ and PMI are surrogates of pressure generated by the respiratory muscles [9, 10, 13, 17], we added *P*_mus_ as another reference standard. Additionally, we could not find studies in which high effort was diagnosed by the criterion of work of breathing. In accordance with the previous studies [12, 13, 17, 25], we set the criteria of high effort as PTP_mus_ per minute higher than 200 cmH_2_O s min^−1^ and *P*_mus_ higher than 10 cmH_2_O, and low effort as PTP_mus_ per minute lower than 50 cmH_2_O s min^−1^ and *P*_mus_ lower than 5 cmH_2_O.

### Accessibility of plateau *P*_aw_ and PMI by end-inspiratory occlusion during PS ventilation

After first introduced by Foti et al. in 1997 [15], several studies investigated the accessibility of plateau *P*_aw_ induced by end-inspiratory occlusion during PS ventilation. In studies conducted by Bellani’s group, the occurrence of unstable plateau *P*_aw_ was approximately 10% [19, 27]. In a secondary analysis of physiologic data from children, 73 of 191 (38%) measurements were excluded due to an inappropriate *P*_aw_ waveform during inspiratory hold [16]. In another retrospective analysis of 40 patients with 227 measurements during PS ventilation, the pattern of *P*_aw_ during an end-inspiratory occlusion cannot assure the absence of expiratory muscle activity [24]. In the present study, we found that 8.6% of end-inspiratory occlusions were immeasurable, mainly due to leaks at high PS levels and continuous respiratory efforts at low support levels. Before the initiation of the study, we conducted training on the performance of airway occlusion and ventilator waveform reading. There are several key points for the successful initiation of end-inspiratory occlusion and obtaining a stable plateau *P*_aw_, including the check of air leak at each support level, and observation of flow-time waveform displayed on the ventilator screen during the occlusion (maintain zero flow). To some extent, these measures may have improved the accessibility of stable plateau *P*_aw_ and the facilitation of PMI measurement. Additionally, all continuous respiratory efforts during occlusion occurred at low support levels, which suggested that irregular plateau *P*_aw_ by end-inspiratory occlusion might have resulted from excessively high effort. In the present study, only 10.7% (3/28) of patients could not tolerate low PS levels (≤ 4 cmH_2_O), and only 6.9% (17/246) of PS levels were categorized as high inspiratory effort (PTP_mus_ per minute > 200 cmH_2_O s min^−1^). The low rate of clinical signs of respiratory distress and low proportion of *P*_es_ measured high inspiratory effort in our group of patients might also explain the low rate of unstable plateau *P*_aw_ during PMI measurements.

In clinical practice, in order to provide accurate measurement of *P*_aw_-based indices, we suggest that at least three airway occlusions, either end-inspiratory or end-expiratory, should be performed, and then averaged to one value.

### Detection of high inspiratory effort

Patients with excessive-high inspiratory effort often exhibit signs of respiratory distress and prompt physicians for emergency treatment [7, 8]. However, a number of patients do not show obvious clinical signs of distress even *P*_es_ measurements indicating high effort. In a study conducted by Pletsch-Assuncao et al., PS titration was performed from 20 cmH_2_O to 2 cmH_2_O [20]. Respiratory distress was observed only at 3.6% (8/219) PS levels. In the present study, downward PS titration was stopped in only three patients (at 4 cmH_2_O) due to respiratory distress; whereas, a high inspiratory effort was diagnosed by the respective criterion of PTP_mus_ per minute and *P*_mus_ at 6.9% to 13.8% PS levels without signs of distress. These results may suggest that other respiratory mechanics instruments should be used as additional tools to detect potential injurious high effort in patients with a high risk of ventilator-induced lung and diaphragmatic injury.

For detecting high effort, our cutoff threshold of *P*_0.1_ (2.0 and 2.2 cmH_2_O, Table 2) was much lower than previous studies (2.7 to 3.5 cmH_2_O by PTP_mus_ per minute ≥ 200 cmH_2_O s min^−1^, and 3.1 cmH_2_O by *P*_mus_ > 10 cmH_2_O) [11, 12, 17]. The same situation was found in the cutoff value of ∆*P*_occ_ to detect high effort, our result (8.4 and 9.2 cmH_2_O, Table 2) was also much lower than the values reported by de Vries et al. (14 and 15 cmH_2_O by the same two criteria used in the present study) [17]. These results’ discrepancy may be due to the difference in severity of respiratory failure between our cohort and above-mentioned investigations. At the study entry, a relatively low PS (median [IQR] of 8 [8–10] cmH_2_O), a normal PaO_2_/FiO_2_ ratio (median [IQR] of 313 [213, 381] mmHg), and a median 7 days of controlled ventilation before the inclusion (Table 1) suggested that most patients in the present study would be ready for a spontaneous breathing trial at the time of inclusion. In this patient population, low levels of PS (2–6 cmH_2_O) might not be able to induce markedly high inspiratory effort. Therefore, although our diagnostic test results of high sensitivity and NPV for the three *P*_aw_-based indices were comparable to previous reports for *P*_0.1_ and ∆*P*_occ_ [12, 13, 17] which suggested their excellent performance of screening and exclusion for high effort, it has to be noted that these results should be interpreted with caution because of a low proportion of high effort during PS titration in our cohort. Regarding the characteristics of patients enrolled in the present study, whether our cutoff values could be used in patients with less severe respiratory failure (e.g., during the weaning phase) requires further investigation.

Additionally, the relatively small sample size of high effort might not be enough to give a conclusion on the diagnostic accuracy of the *P*_aw_-based indices in the present study. Especially for the PMI in detecting high inspiratory effort, to the best of our knowledge, no study has been performed to test the diagnostic accuracy of PMI. This needs further study.

### Detection of low inspiratory effort

Recent evidence showed that low inspiratory effort, usually induced by over-assistance and sedation, could result in respiratory muscle atrophy and dysfunction [2]. Due to the lack of easily accessible evaluation tools, the low inspiratory effort seems to be underestimated [20, 28]. As a reliable measure of respiratory drive, *P*_0.1_ has been investigated for the assessment of low effort [12]. Many modern ventilators integrate the function of automated *P*_0.1_ measurement, which facilitates easy bedside use [29]. Our results showed a good diagnostic performance for *P*_0.1_ to detect low effort with cutoff value of 1.1 cmH_2_O by PTP_mus_ and *P*_mus_ criteria (Table 3), which were comparable to the study conducted by Telias et al. using PTP_mus_ criterion (1.0 cmH_2_O) [12] and de Vries et al. using *P*_mus_ criterion (1.3 cmH_2_O) [17]. Our threshold of ∆*P*_occ_ for detecting low effort (6.3 cmH_2_O) was slightly lower than the result (9 cmH_2_O) reported by de Vries et al. with the same *P*_mus_ criterion (< 5 cmH_2_O) [17]. Up to now, we have not found a study to investigate the use of PMI to detect low inspiratory effort.

### Clinical implications

In the past decade, the role of insufficient and excessive inspiratory effort in ventilator-associated lung and diaphragm injuries has attracted clinical attention [2, 3]. Bedside assessment of inspiratory effort is the most important step to concomitantly protect both the lung and the diaphragm during assisted ventilation [5]. As a reference standard for effort evaluation, *P*_es_ is not routinely used in clinical practice [7, 8]. In the present study, we demonstrated the accuracy of three *P*_aw_-based indices to detect low and high inspiratory effort. These indices could be used as a supplement to clinical observation (e.g., sedation assessment) and an indication for further direct clinical monitoring (e.g., *P*_es_) to confirm the low and high effort.

In accordance with previous studies [11–14, 17], we found that *P*_0.1_ and ∆*P*_occ_ can be easily and reliably obtained. Although earlier studies have shown a relatively high rate of inaccessibility of PMI due to unstable plateau *P*_aw_ during end-inspiratory occlusion [16, 24], a standardized performance of airway occlusion and training (mentioned above) improved the measurement of PMI in the present study. In addition to effort assessment, PMI also has the advantage of simultaneously obtaining airway driving pressure and respiratory compliance. Our reference intervals analysis showed that the percentage of conditions in the “grey zone” (defined as conditions outside of the lower to upper limit range of reference intervals) ranged from low to high as *P*_0.1_ (4.3% and 3.1% by the respective PTP_mus_ per minute and *P*_mus_ criterion) < ∆*P*_occ_ (6.5% and 9.2%) < PMI (9.7% and 12.3%) (Additional file 1**:** Table S5). Based on our data, we suggest that if the physician only wants to evaluate the effort, *P*_0.1_ and ∆*P*_occ_ are preferred in ventilator with and without the function of automated *P*_0.1_ measurement, respectively. However, if one wishes to evaluate compliance simultaneously, applying PMI may be a good choice.

### Limitations

This study has limitations. First, although we enrolled acute respiratory failure patients during the first 24 h undergoing PS ventilation, the patients had received a relatively long duration of controlled mechanical ventilation. These patients had relatively stable oxygenation and respiratory mechanics (Table 1). Our results might not be applicable to other populations, especially those during the acute phase of respiratory failure with a high risk of excessive inspiratory effort. Additionally, we excluded patients with COPD because some patients could not tolerate low PS levels (2 to 8 cmH_2_O) during our pilot test. However, the risk of over-assistance and diaphragmatic disuse atrophy is particularly high in COPD patients, and further study is needed to clarify the usefulness of *P*_aw_-based indices for effort monitoring in this population. Second, although we used the inspiratory effort criteria according to previous reports [12, 17], a universally accepted definition is still lacking. Third, although a stable diagnostic model was confirmed by internal cross-validation, our results need further external validation. Fourth, in the present study, sequential downward PS titration was used but not a random selection of support level because we wanted to avoid possible termination of evaluation due to fatigue resulting from low initial PS levels. This strategy was applied in the previous study for the same reason [19]. Fifth, end-inspiratory occlusion cannot be performed during PS mode in some ventilators, which may limit the clinical use of PMI.

## Conclusions

The three *P*_aw_-based indices, *P*_0.1_, ∆*P*_occ_, and PMI, are accurate in detecting low inspiratory effort during PS ventilation. Non-invasive and easily accessible characteristics support their potential bedside use for avoiding over-assistance. Although our results also showed good performance of the three *P*_aw_-based indices in detecting high effort, it deserves further study because a low incidence of high effort was induced in our cohort.

### Supplementary Information

**Additional file 1: Figure S1.** Inspiratory muscle pressure, inspiratory muscle pressure–time product per minute, and airway pressure-based indices during downward pressure support titration. **Table S1.** Comparison of parameters in different inspiratory effort groups using the criterion of inspiratory muscle pressure–time product. **Table S2.** Comparison of parameters in different inspiratory effort groups using the criterion of inspiratory muscle pressure. **Table S3.** Tenfold cross-validation of airway pressure-based indices for diagnosis of high effort. **Table S4.** Tenfold cross-validation of airway pressure-based indices for diagnosis of low effort. **Table S5.** Lower and upper limits of reference intervals of airway pressure-based indices classified by inspiratory muscle pressure per minute or inspiratory muscle pressure.

## Data Availability

The datasets used and/or analyzed during the current study are available from the corresponding author on reasonable request.

## Abbreviations

APACHE: Acute Physiology, Age and Chronic Health Evaluation
AUC: Area under the curve
CI: Confidence interval
COPD: Chronic obstructive pulmonary diseases
FiO_2_: Inspired oxygen fraction
IQR: Interquartile range
NPV: Negative predictive values
PaCO_2_: Partial pressure of carbon dioxide in arterial blood
PaO_2_: Partial pressure of oxygen in arterial blood
*P*_aw_: Airway pressure
PEEP: Positive end-expiratory pressure
*P*_es_: Esophageal pressure
PMI: Pressure muscle index
*P*_mus_: Inspiratory muscle pressure
PPV: Positive predictive values
PS: Pressure support
PTP_mus_: Inspiratory muscle pressure–time product
*P*_0.1_: Airway occlusion pressure
∆*P*_occ_: The swing in airway pressure generated by the patient’s respiratory effort
RASS: Richmond Agitation Sedation Scale
ROC: Receiver operating characteristic
STARD: Standards for Reporting of Diagnostic Accuracy

## References

[1] Brochard LJ, Lellouche F, Tobin MJ (2013). Pressure support ventilation. Principles and practice of mechanical ventilation.

[2] Goligher EC, Jonkman AH, Dianti J, Vaporidi K, Beitler JR, Patel BK, Yoshida T, Jaber S, Dres M, Mauri T, Bellani G, Demoule A, Brochard L, Heunks L (2020). Clinical strategies for implementing lung and diaphragm-protective ventilation: avoiding insufficient and excessive effort. Intensive Care Med.

[3] Brochard L, Slutsky A, Pesenti A (2017). Mechanical ventilation to minimize progression of lung injury in acute respiratory failure. Am J Respir Crit Care Med.

[4] Brochard L, Telias I (2018). Bedside detection of overassistance during pressure support ventilation. Crit Care Med.

[5] Goligher EC, Dres M, Patel BK, Sahetya SK, Beitler JR, Telias I, Yoshida T, Vaporidi K, Grieco DL, Schepens T, Grasselli G, Spadaro S, Dianti J, Amato M, Bellani G, Demoule A, Fan E, Ferguson ND, Georgopoulos D, Guerin C, Khemani RG, Laghi F, Mercat A, Mojoli F, Ottenheijm CAC, Jaber S, Heunks L, Mancebo J, Mauri T, Pesenti A, Brochard L (2020). Lung- and Diaphragm-protective ventilation. Am J Respir Crit Care Med.

[6] Goligher EC, Brochard LJ, Reid WD, Fan E, Saarela O, Slutsky AS, Kavanagh BP, Rubenfeld GD, Ferguson ND (2019). Diaphragmatic myotrauma: a mediator of prolonged ventilation and poor patient outcomes in acute respiratory failure. Lancet Respir Med.

[7] de Vries H, Jonkman A, Shi ZH, Spoelstra-de Man A, Heunks L (2018). Assessing breathing effort in mechanical ventilation: physiology and clinical implications. Ann Transl Med.

[8] Telias I, Spadaro S (2020). Techniques to monitor respiratory drive and inspiratory effort. Curr Opin Crit Care.

[9] Akoumianaki E, Maggiore SM, Valenza F, Bellani G, Jubran A, Loring SH, Pelosi P, Talmor D, Grasso S, Chiumello D, Guerin C, Patroniti N, Ranieri VM, Gattinoni L, Nava S, Terragni PP, Pesenti A, Tobin M, Mancebo J, Brochard L (2014). The application of esophageal pressure measurement in patients with respiratory failure. Am J Respir Crit Care Med.

[10] Mauri T, Yoshida T, Bellani G, Goligher EC, Carteaux G, Rittayamai N, Mojoli F, Chiumello D, Piquilloud L, Grasso S, Jubran A, Laghi F, Magder S, Pesenti A, Loring S, Gattinoni L, Talmor D, Blanch L, Amato M, Chen L, Brochard L, Mancebo J, PLeUral pressure working Group (2016). Esophageal and transpulmonary pressure in the clinical setting: meaning, usefulness and perspectives. Intensive Care Med.

[11] Rittayamai N, Beloncle F, Goligher EC, Chen L, Mancebo J, Richard JM, Brochard L (2017). Effect of inspiratory synchronization during pressure-controlled ventilation on lung distension and inspiratory effort. Ann Intensive Care.

[12] Telias I, Junhasavasdikul D, Rittayamai N, Piquilloud L, Chen L, Ferguson ND, Goligher EC, Brochard L (2020). Airway occlusion pressure as an estimate of respiratory drive and inspiratory effort during assisted ventilation. Am J Respir Crit Care Med.

[13] Bertoni M, Telias I, Urner M, Long M, Del Sorbo L, Fan E, Sinderby C, Beck J, Liu L, Qiu H, Wong J, Slutsky AS, Ferguson ND, Brochard LJ, Goligher EC (2019). A novel non-invasive method to detect excessively high respiratory effort and dynamic transpulmonary driving pressure during mechanical ventilation. Crit Care.

[14] Roesthuis L, van den Berg M, van der Hoeven H (2021). Non-invasive method to detect high respiratory effort and transpulmonary driving pressures in COVID-19 patients during mechanical ventilation. Ann Intensive Care.

[15] Foti G, Cereda M, Banfi G, Pelosi P, Fumagalli R, Pesenti A (1997). End-inspiratory airway occlusion: a method to assess the pressure developed by inspiratory muscles in patients with acute lung injury undergoing pressure support. Am J Respir Crit Care Med.

[16] Kyogoku M, Shimatani T, Hotz JC, Newth CJL, Bellani G, Takeuchi M, Khemani RG (2021). Direction and magnitude of change in plateau from peak pressure during inspiratory holds can identify the degree of spontaneous effort and elastic workload in ventilated patients. Crit Care Med.

[17] de Vries HJ, Tuinman PR, Jonkman AH, Liu L, Qiu H, Girbes ARJ, Zhang Y, de Man AME, de Grooth HJ, Heunks L (2023). Performance of noninvasive airway occlusion maneuvers to assess lung stress and diaphragm effort in mechanically ventilated critically ill patients. Anesthesiology.

[18] Cohen JF, Korevaar DA, Altman DG, Bruns DE, Gatsonis CA, Hooft L, Irwig L, Levine D, Reitsma JB, de Vet HC, Bossuyt PM (2016). STARD 2015 guidelines for reporting diagnostic accuracy studies: explanation and elaboration. BMJ Open.

[19] Bellani G, Grassi A, Sosio S, Gatti S, Kavanagh BP, Pesenti A, Foti G (2019). Driving pressure is associated with outcome during assisted ventilation in acute respiratory distress syndrome. Anesthesiology.

[20] Pletsch-Assuncao R, Caleffi Pereira M, Ferreira JG, Cardenas LZ, de Albuquerque ALP, de Carvalho CRR, Caruso P (2018). Accuracy of invasive and noninvasive parameters for diagnosing ventilatory overassistance during pressure support ventilation. Crit Care Med.

[21] Luo XY, He X, Zhou YM, Wang YM, Chen JR, Chen GQ, Li HL, Yang YL, Zhang L, Zhou JX (2020). Patient-ventilator asynchrony in acute brain-injured patients: a prospective observational study. Ann Intensive Care.

[22] Alberti A, Gallo F, Fongaro A, Valenti S, Rossi A (1995). P0.1 is a useful parameter in setting the level of pressure support ventilation. Intensive Care Med.

[23] Sassoon CS, Mahutte CK, Te TT, Simmons DH, Light RW (1988). Work of breathing and airway occlusion pressure during assist-mode mechanical ventilation. Chest.

[24] Soundoulounaki S, Akoumianaki E, Kondili E, Pediaditis E, Prinianakis G, Vaporidi K, Georgopoulos D (2020). Airway pressure morphology and respiratory muscle activity during end-inspiratory occlusions in pressure support ventilation. Crit Care.

[25] Albani F, Fusina F, Ciabatti G, Pisani L, Lippolis V, Franceschetti ME, Giovannini A, di Mussi R, Murgolo F, Rosano A, Grasso S, Natalini G (2021). Flow Index accurately identifies breaths with low or high inspiratory effort during pressure support ventilation. Crit Care.

[26] Ozarda Y, Higgins V, Adeli K (2018). Verification of reference intervals in routine clinical laboratories: practical challenges and recommendations. Clin Chem Lab Med.

[27] Bellani G, Grasselli G, Teggia-Droghi M, Mauri T, Coppadoro A, Brochard L, Pesenti A (2016). Do spontaneous and mechanical breathing have similar effects on average transpulmonary and alveolar pressure? A clinical crossover study. Crit Care.

[28] Al-Bassam W, Dade F, Bailey M, Eastwood G, Osawa E, Eyeington C, Anstey J, Yi G, Ralph J, Kakho N, Kurup V, Licari E, King EC, Knott C, Chimunda T, Smith J, Subramaniam A, Reddy M, Green C, Parkin G, Shehabi Y, Bellomo R (2019). "Likely overassistance" during invasive pressure support ventilation in patients in the intensive care unit: a multicentre prospective observational study. Crit Care Resusc.

[29] Beloncle F, Piquilloud L, Olivier PY, Vuillermoz A, Yvin E, Mercat A, Richard JC (2019). Accuracy of P0.1 measurements performed by ICU ventilators: a bench study. Ann Intensive Care.

