# Supplementary material for: Use of airway pressure-based indices to detect high and low inspiratory effort during pressure support ventilation: a diagnostic accuracy study
Source: Ann Intensive Care. 2023 Nov 13;13:111. doi: 10.1186/s13613-023-01209-7 (PMC10643759; doi:10.1186/s13613-023-01209-7)
Supplement: Supplementary file 1 — Additional file 1: Figure S1. Inspiratory muscle pressure, inspiratory muscle pressure–time product per minute, and airway pressure-based indices during downward pressure support titration. Table S1. Comparison of parameters in different inspiratory effort groups using the criterion of inspiratory muscle pressure–time product. Table S2. Comparison of parameters in different inspiratory effort groups using the criterion of inspiratory muscle pressure. Table S3. Tenfold cross-validation of airway pressure-based indices for diagnosis of high effort. Table S4. Tenfold cross-validation of airway pressure-based indices for diagnosis of low effort. Table S5. Lower and upper limits of reference intervals of airway pressure-based indices classified by inspiratory muscle pressure per minute or inspiratory muscle pressure. [file 13613_2023_1209_MOESM1_ESM.pdf]

Use of airway pressure-based indices to detect high and low inspiratory effort during pressure support ventilation: a diagnostic accuracy study

Yan-Lin Yang, Yang Liu, Ran Gao, De-Jing Song, Yi-Min Zhou, Ming-Yue Miao, Wei Chen, Shu-Peng Wang, Yue-Fu Wang, Linlin Zhang, Jian-Xin Zhou

Additional file 1

Contents

- ☐ **Figure S1.** Inspiratory muscle pressure, inspiratory muscle pressure-time product per minute, and airway pressure-based indices during downward pressure support titration
- ☐ **Table S1.** Comparison of parameters in different inspiratory effort groups using the criterion of inspiratory muscle pressure-time product
- ☐ **Table S2.** Comparison of parameters in different inspiratory effort groups using the criterion of inspiratory muscle pressure
- ☐ **Table S3.** Ten-fold cross-validation of airway pressure-based indices for diagnosis of high effort
- ☐ **Table S4.** Ten-fold cross-validation of airway pressure-based indices for diagnosis of low effort
- ☐ **Table S5.** Lower and upper limits of reference intervals of airway pressure-based indices classified by inspiratory muscle pressure per minute or inspiratory muscle pressure

Figure S1. Inspiratory muscle pressure, inspiratory muscle pressure-time product per minute, and airway pressure-based indices during downward pressure support titration

A

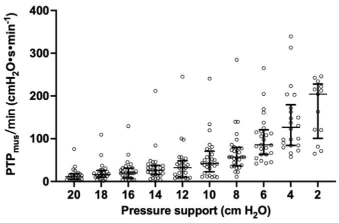

	20	18	16	14	12	10	8	6	4	2
20	-									
18	ns	-								
16	ns	ns	-							
14	ns	ns	ns	-						
12	ns	ns	ns	ns	-					
10	0.014	ns	ns	ns	ns	-				
8	<0.001	0.002	0.009	ns	ns	ns	-			
6	<0.001	<0.001	<0.001	<0.001	0.001	ns	ns	-		
4	<0.001	<0.001	<0.001	<0.001	<0.001	0.003	ns	ns	-	
2	<0.001	<0.001	<0.001	<0.001	<0.001	<0.001	ns	ns	ns	-

B

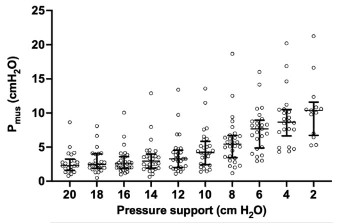

	20	18	16	14	12	10	8	6	4	2
20	-									
18	ns	-								
16	ns	ns	-							
14	ns	ns	ns	-						
12	ns	ns	ns	ns	-					
10	ns	ns	ns	ns	ns	-				
8	0.006	0.019	0.010	ns	ns	ns	-			
6	<0.001	<0.001	<0.001	<0.001	0.002	ns	ns	-		
4	<0.001	<0.001	<0.001	<0.001	<0.001	0.010	ns	ns	-	
2	<0.001	<0.001	<0.001	<0.001	<0.001	0.011	ns	ns	ns	-

C

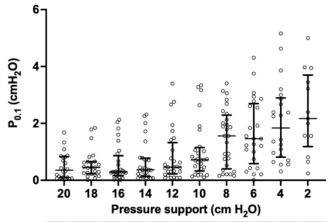

	20	18	16	14	12	10	8	6	4	2
20	-									
18	ns	-								
16	ns	ns	-							
14	ns	ns	ns	-						
12	ns	ns	ns	ns	-					
10	ns	ns	ns	ns	ns	-				
8	ns	ns	ns	ns	ns	ns	-			
6	0.014	ns	0.013	0.034	ns	ns	ns	-		
4	<0.001	0.001	<0.001	<0.001	0.008	ns	ns	ns	-	
2	<0.001	<0.001	<0.001	<0.001	0.001	0.022	ns	ns	ns	-

D

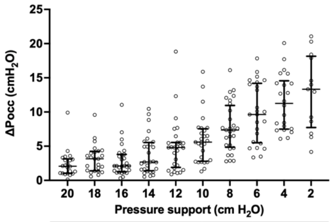

	20	18	16	14	12	10	8	6	4	2
20	-									
18	ns	-								
16	ns	ns	-							
14	ns	ns	ns	-						
12	ns	ns	ns	ns	-					
10	ns	0.017	ns	ns	ns	-				
8	<0.001	<0.001	<0.001	0.019	ns	ns	-			
6	<0.001	<0.001	<0.001	<0.001	0.010	ns	ns	-		
4	<0.001	<0.001	<0.001	<0.001	<0.001	0.012	ns	ns	-	
2	<0.001	<0.001	<0.001	<0.001	<0.001	0.009	ns	ns	ns	-

E

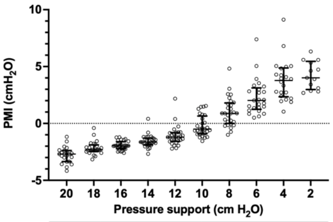

	20	18	16	14	12	10	8	6	4	2
20	-									
18	ns	-								
16	ns	ns	-							
14	ns	ns	ns	-						
12	0.007	ns	ns	ns	-					
10	<0.001	<0.001	0.007	ns	ns	-				
8	<0.001	<0.001	<0.001	0.002	ns	ns	-			
6	<0.001	<0.001	<0.001	<0.001	<0.001	ns	ns	-		
4	<0.001	<0.001	<0.001	<0.001	<0.001	0.004	ns	ns	-	
2	<0.001	<0.001	<0.001	<0.001	<0.001	0.001	ns	ns	ns	-

Data are shown as median and interquartile range. Results of pairwise post-hoc analysis are also shown.

Downward pressure support adjustment resulted in a significant change in inspiratory muscle pressure-time product per minute (A), inspiratory muscle pressure (B),and airway pressure-based indices (C, D, and E) ($p < 0.001$).

ΔP_{occ} the maximal negative swing of airway pressure against end-expiratory airway occlusion, $P_{0.1}$ the negative swing of airway pressure against end-expiratory airway occlusion at first 100ms, *PMI* pressure muscle index, P_{mus} inspiratory muscle pressure, PTP_{mus}/min inspiratory muscle pressure-time product per minute.

Table S1. Comparison of parameters in different inspiratory effort groups using the criterion of inspiratory muscle pressure-time product

	Low inspiratory effort n = 136	Intermediate inspiratory effort n = 93	High inspiratory effort n = 17	*P*
Pressure support (cmH_2O) [b]	14 (12, 18)	6 (4, 8)	4 (2, 7)	<0.001
PTP_{mus} per minute (cmH_2O·s/min) [a]	19.2 (9.6, 33.5)	90.0 (69.6, 122.4)	234.4 (212.1, 255.6)	<0.001
ΔP_{es} (cmH_2O) [a]	2.6 (1.9, 3.7)	7.6 (5.6, 9.4)	13.4 (12.1, 16.7)	<0.001
P_{mus} (cmH_2O) [a]	0.8 (0.4, 1.3)	4.3 (2.5, 6.1)	8.8 (6.3, 13.4)	<0.001
PEEPi (cmH_2O) [a]	0 (0, 1)	1 (0, 1)	2 (1, 3)	<0.001
VTi/PBW (mL/kg) [b]	12.6 (9.8, 15.1)	8.4 (6.8, 10.9)	7.8 (6.6, 10.5)	<0.001
Respiratory rate (bpm) [b]	12 (10, 15)	19 (15, 22)	22 (19, 24)	<0.001
V_E (L/min)	8.7 (6.6, 10.7)	8.8 (7.0, 13.4)	9.3 (8.2, 13.2)	0.077
$P_{0.1}$ (cmH_2O) [a]	0.3 (0.2, 0.8)	1.6 (0.8, 2.7)	3.3 (2.3, 3.9)	<0.001
ΔP_{occ} (cmH_2O) [a]	2.8 (1.4, 4.8)	9.1 (6.4, 12.3)	14.7 (11.6, 18.2)	<0.001
PMI (cmH_2O) [b]	-1.7 (-2.3, -1.2)	2.0 (0.8, 3.3)	4.6 (3.3, 6.0)	<0.001

Data are shown as median (interquartile range)

The low, intermediate, and high inspiratory effort was pre-defined as inspiratory muscle pressure-time product per minute of < 50, 50–200, and > 200 $cmH_2O \cdot s \cdot min^{-1}$, respectively.

ΔP_{es} tidal swing of esophageal pressure, P_{mus} inspiratory muscle pressure, PTP_{mus} inspiratory muscle pressure-time product, *PEEPi* intrinsic positive end-expiratory pressure, *VTi* inspiratory tidal volume, *PBW* ideal body weight, $P_{0.1}$ airway occlusion pressure, ΔP_{occ} negative swing of airway pressure against end-expiratory airway occlusion, *PMI* pressure muscle index, V_E minute ventilation

[a] $P < 0.05$ pairwise comparison among the three groups

[b] $P < 0.05$ low effort group compared to intermediate and high effort groups

Table S2. Comparison of parameters in different inspiratory effort groups using the criterion of inspiratory muscle pressure

	Low inspiratory effort n = 146	Intermediate inspiratory effort n = 65	High inspiratory effort n = 35	*P*
Pressure support (cmH_2O) [a]	14 (10, 18)	8 (4, 10)	4 (2, 6)	<0.001
PTP_{mus} per minute ($cmH_2O·s/min$) [a]	20.0 (10.1, 37.1)	84.8 (69.6, 111.7)	180.1 (129.2, 234.4)	<0.001
ΔP_{es} (cmH_2O) [a]	2.7 (1.9, 3.8)	7.2 (6.2, 8.5)	12.0 (10.6, 13.6)	<0.001
P_{mus} (cmH_2O) [a]	0.8 (0.4, 1.5)	3.9 (2.5, 5.6)	7.6 (6.1, 9.8)	<0.001
PEEPi (cmH_2O) [a]	0 (0, 1)	1 (0, 1)	1 (1, 3)	<0.001
VTi/PBW (mL/kg) [b]	11.5 (8.9, 14.9)	7.4 (8.6, 11.3)	7.8 (6.5, 11.3)	<0.001
Respiratory rate (bpm) [b]	12 (10, 16)	17 (14, 22)	21 (18, 23)	<0.001
V_E (L/min)	8.6 (6.6, 10.7)	9.0 (7.3, 13.4)	9.3 (8.0, 14.2)	0.020
$P_{0.1}$ (cmH_2O) [a]	0.4 (0.2, 0.7)	1.7 (0.8, 2.4)	3.0 (2.3, 3.8)	<0.001
ΔP_{occ} (cmH_2O) [a]	3.1 (1.4, 4.9)	9.0 (6.5, 11.4)	14.2 (11.3, 17.7)	<0.001
PMI (cmH_2O) [a]	-1.6 (-2.2, -0.8)	1.8 (0.0, 2.8)	4.2 (3.0, 5.0)	<0.001

Data are shown as median (interquartile range)

The low, intermediate, and high inspiratory effort was pre-defined as inspiratory muscle pressure of < 5, 5–10, and > 10 cmH_2O, respectively.

ΔP_{es} tidal swing of esophageal pressure, P_{mus} inspiratory muscle pressure, PTP_{mus} inspiratory muscle pressure-time product, *PEEPi* intrinsic positive end-expiratory pressure, *VTi* inspiratory tidal volume, *PBW* ideal body weight, $P_{0.1}$ airway occlusion pressure, ΔP_{occ} negative swing of airway pressure against end-expiratory airway occlusion, *PMI* pressure muscle index, V_E minute ventilation

[a] $P < 0.05$ pairwise comparison among the three groups

[b] $P < 0.05$ low effort group compared to intermediate and high effort groups

[c] $P < 0.05$ low effort group compared to high effort groups

Table S3. Ten-fold cross-validation of airway pressure-based indices for diagnosis of high effort

	AUROC	Cutoff	Sensitivity	Specificity	PPV	NPV
Classified by PTP_{mus} per minute						
$P_{0.1}$	0.94 ± 0.01	2.2 ± 0.0	1.00 ± 0.00	0.86 ± 0.01	0.32± 0.02	1.00 ± 0.00
ΔP_{occ}	0.93 ± 0.01	8.6 ± 0.3	1.00 ± 0.00	0.76 ± 0.01	0.24 ± 0.01	1.00 ± 0.00
PMI	0.93 ± 0.01	2.3 ± 0.6	0.88 ± 0.04	0.82 ± 0.04	0.28 ± 0.06	0.99 ± 0.00
Classified by P_{mus}						
$P_{0.1}$	0.95 ± 0.01	2.0 ± 0.1	0.91 ± 0.02	0.88 ± 0.02	0.54 ± 0.10	0.98 ± 0.00
ΔP_{occ}	0.93 ± 0.01	9.3 ± 0.1	0.94 ± 0.01	0.85 ± 0.01	0.50 ± 0.02	0.99 ± 0.00
PMI	0.93 ± 0.01	3.0 ± 0.2	0.79 ± 0.03	0.93 ± 0.02	0.66 ± 0.06	0.96 ± 0.00

Data are shown as mean ± standard deviation.
$P_{0.1}$ the negative swing of airway pressure against end-expiratory airway occlusion at first 100ms, ΔP_{occ} the maximal negative swing of Paw against end-expiratory airway occlusion, *PMI* pressure muscle index, P_{mus} inspiratory muscle pressure, *PTPmus* inspiratory muscle pressure-time product, *AUROC* area under the receiver-operating-characteristics curve, *PPV* positive predictive value, *NPV* negative predictive value

Table S4. Ten-fold cross-validation of airway pressure-based indices for diagnosis of low effort

	AUROC	Cutoff	Sensitivity	Specificity	PPV	NPV
Classified by PTP_{mus} per minute						
$P_{0.1}$	0.87 ± 0.01	1.1 ± 0.1	0.88 ± 0.02	0.73 ± 0.02	0.80 ± 0.01	0.83 ± 0.02
ΔP_{occ}	0.93 ± 0.01	5.8 ± 0.2	0.87 ± 0.01	0.85 ± 0.02	0.88 ± 0.01	0.84 ± 0.01
PMI	0.95 ± 0.01	0.0 ± 0.3	0.94 ± 0.02	0.88 ± 0.03	0.91 ± 0.02	0.93 ± 0.02
Classified by P_{mus}						
$P_{0.1}$	0.90 ± 0.01	1.2 ± 0.1	0.89 ± 0.01	0.79 ± 0.02	0.86 ± 0.01	0.83 ± 0.02
ΔP_{occ}	0.94 ± 0.01	6.2 ± 0.3	0.87 ± 0.02	0.87 ± 0.02	0.90 ± 0.01	0.82 ± 0.02
PMI	0.89 ± 0.02	0.4 ± 0.5	0.89 ± 0.04	0.83 ± 0.04	0.88 ± 0.02	0.84 ± 0.05

Data are shown as mean ± standard deviation.
$P_{0.1}$ the negative swing of airway pressure against end-expiratory airway occlusion at first 100ms, ΔP_{occ} the maximal negative swing of Paw against end-expiratory airway occlusion, *PMI* pressure muscle index, P_{mus} inspiratory muscle pressure, *PTPmus* inspiratory muscle pressure-time product, *AUROC* area under the receiver-operating-characteristics curve, *PPV* positive predictive value, *NPV* negative predictive value

Table S5. Lower and upper limits of reference intervals of airway pressure-based indices classified by inspiratory muscle pressure per minute or inspiratory muscle pressure

Indices	median	Lower and upper limits of reference intervals		condition in "grey zone"
		lower limit	upper limit	n = 93
Classified by PTP_{mus} per minute				
$P_{0.1}$ (cmH_2O)	1.6	0.2 (0.2, 0.4)	3.4 (3.1, 4.0)	4.3%
ΔPocc (cmH_2O)	9.1	2.6 (1.6, 3.6)	15.9 (14.8, 17.0)	6.5%
PMI (cmH_2O)	2.0	-1.1 (-1.6, -0.5)	5.2 (4.7, 5.6)	9.7%
Classified by P_{mus}				
$P_{0.1}$ (cmH_2O)	1.7	0.2 (0.2, 0.4)	3.1 (2.8, 3.3)	3.1%
ΔPocc (cmH_2O)	8.9	3.5 (2.5, 4.5)	14.5 (13.4, 15.4)	9.2%
PMI (cmH_2O)	1.8	-1.7 (-2.6 -0.9)	5.3 (4.6, 5.9)	12.3%

95% confidence intervals are shown in parentheses for diagnostic performance measures.

P_{mus} inspiratory muscle pressure, *PTPmus* inspiratory muscle pressure-time product, $P_{0.1}$ airway occlusion pressure, *ΔPocc* negative swing of airway pressure against end-expiratory airway occlusion, *PMI* pressure muscle index
